# Supplementary material for: Leisure Time Use and Adolescent Mental Well-Being: Insights from the COVID-19 Czech Spring Lockdown
Source: Int J Environ Res Public Health. 2021 Dec 5;18(23):12812. doi: 10.3390/ijerph182312812 (PMC8657078; doi:10.3390/ijerph182312812)
Supplement: Supplementary file 1 [file ijerph-18-12812-s001.zip › Supplementary Table S1 lifesat.pdf]

Supplementary Table S1 Gender-stratified associations between different leisure components, school, and life satisfaction

	Boys					Girls				
**Model**	**B**	**SE**	**ß**	**t**	**p**	**B**	**SE**	**ß**	**t**	**p**
(Intercept)	8.069	0.456		17.71	<0.001	8.65	0.444		19.49	<0.001
Age	**−0.078**	**0.03**	**−0.075**	**−2.578**	**0.01**	**−0.161**	**0.03**	**−0.138**	**−5.433**	**<0.001**
Leisure (hrs)	**0.137**	**0.027**	**0.153**	**5.093**	**<0.001**	**0.169**	**0.026**	**0.173**	**6.59**	**<0.001**
Schoolwork (hrs)	0.039	0.033	0.035	1.16	0.246	0.048	0.031	0.041	1.527	0.127
Perceived more leisure †	0.126	0.122	0.035	1.033	0.302	**0.243**	**0.116**	**0.062**	**2.106**	**0.035**
Perceived less leisure †	**−0.324**	**0.163**	**−0.068**	**−1.991**	**0.047**	−0.261	0.162	−0.048	−1.617	0.106
Perceived more schoolwork †	0.052	0.119	0.015	0.436	0.663	−0.056	0.118	−0.015	−0.477	0.634
Perceived less schoolwork †	−0.035	0.135	−0.009	−0.26	0.795	−0.098	0.136	−0.022	−0.719	0.472
Social active leisure	**0.17**	**0.058**	**0.101**	**2.92**	**0.004**	**0.12**	**0.058**	**0.062**	**2.054**	**0.04**
Cultural creative leisure	−0.066	0.057	−0.034	−1.159	0.247	−0.03	0.047	−0.017	−0.643	0.521
Idle leisure	**−0.17**	**0.053**	**−0.099**	**−3.239**	**0.001**	**−0.205**	**0.052**	**−0.109**	**−3.931**	**<0.001**
Sports and physical activity	**0.233**	**0.059**	**0.138**	**3.92**	**<0.001**	**0.17**	**0.059**	**0.089**	**2.865**	**0.004**
Electronic media use	−0.055	0.054	−0.032	−1.014	0.311	**−0.209**	**0.053**	**−0.112**	**−3.927**	**<0.001**

† Those who perceived no change in the amount of their leisure time or time spent on schoolwork served as a reference group.
