# Supplementary material for: Leisure Time Use and Adolescent Mental Well-Being: Insights from the COVID-19 Czech Spring Lockdown
Source: Int J Environ Res Public Health. 2021 Dec 5;18(23):12812. doi: 10.3390/ijerph182312812 (PMC8657078; doi:10.3390/ijerph182312812)
Supplement: Supplementary file 1 [file ijerph-18-12812-s001.zip › Supplementary Table S2 WHO5 wellbeing.pdf]

Supplementary Table S2 Gender-stratified associations between different leisure components, school, and WHO-5 Well-being Index

	Boys					Girls				
**Model**	**B**	**SE**	**ß**	**t**	**p**	**B**	**SE**	**ß**	**t**	**p**
(Intercept)	59.714	6.268		9.526	<0.001	67.855	5.816		11.667	<0.001
Age	**−0.833**	**0.417**	**−0.058**	**−1.997**	**0.046**	**−2.099**	**0.391**	**−0.132**	**−5.361**	**<0.001**
Leisure (hrs)	**2.129**	**0.371**	**0.17**	**5.733**	**<0.001**	**2.555**	**0.336**	**0.194**	**7.604**	**<0.001**
Schoolwork (hrs)	0.406	0.465	0.026	0.873	0.383	0.141	0.413	0.009	0.342	0.732
Perceived more leisure †	2.242	1.686	0.045	1.329	0.184	**4.227**	**1.531**	**0.079**	**2.761**	**0.006**
Perceived less leisure †	−3.779	2.245	−0.057	−1.683	0.093	−3.612	2.126	−0.049	−1.699	0.09
Perceived more schoolwork †	1.188	1.646	0.025	0.722	0.47	−0.361	1.552	−0.007	−0.232	0.816
Perceived less schoolwork †	0.752	1.859	0.014	0.405	0.686	−1.677	1.792	−0.028	−0.936	0.349
Social active leisure	**3.854**	**0.808**	**0.165**	**4.772**	**<0.001**	**3.581**	**0.769**	**0.138**	**4.657**	**<0.001**
Cultural creative leisure	0.042	0.787	0.002	0.053	0.957	0.182	0.624	0.007	0.292	0.77
Idle leisure	**−3.77**	**0.73**	**−0.157**	**−5.163**	**<0.001**	**−4.093**	**0.691**	**−0.16**	**−5.925**	**<0.001**
Sports and physical activity	**4.566**	**0.822**	**0.196**	**5.552**	**<0.001**	**3.493**	**0.79**	**0.134**	**4.42**	**<0.001**
Electronic media use	1.332	0.743	0.056	1.792	0.073	**−2.234**	**0.711**	**−0.087**	**−3.141**	**0.002**

† Those who perceived no change in the amount of their leisure time or time spent on schoolwork served as a reference group.
